# Exploring Extreme Weather and Recess Policies, Practices, and Procedures in the Canadian Context

**DOI:** 10.3390/ijerph20010814

**Published:** 2023-01-01

**Authors:** Brenton L. G. Button, Gina Martin

**Affiliations:** 1 Faculty of Education, University of Winnipeg, Winnipeg, MB R3B 2E9, Canada; 2 Faculty of Health Disciplines, Athabasca University, Athabasca, AB T9S 3A3, Canada; 3 Department of Geography and Environment, Western University, London, ON N6A 3K7, Canada

**Keywords:** recess, policy, well-being, weather, child

## Abstract

The purpose of this study was to explore the different policies, practices, and procedures that are used on weather and recess in the Canadian context. Fifty school websites were examined, and ten key informants were interviewed. Policies, practices, and procedures from school websites were downloaded, and interviews were transcribed verbatim and analyzed using a qualitative content analysis. Fourteen schools had an outwardly facing policy, practice, or procedure for weather and recess. Cold temperatures were the most often cited reason for modifying recess to be indoors, with temperatures ranging from −20 to −40 for complete indoor recess. Precipitation was only found in four online documents but was mentioned as a reason to modify recess by all key informants. Additionally, key informants discussed variability in how recess policies, practices, and procedures were followed. The findings of this study illustrate inconsistencies in both formal and informal school weather and recess policies. With outdoor recess providing numerous opportunities to improve various domains of well-being, it is pertinent to understand the conditions on which it is being modified.

## 1. Introduction

In Canada, children spend approximately 30 h per week in the school environment [1]. Most of this time is spent working in a structured or semi-structured environment in the confines of the classroom, which is typically designed for sitting or low-intensity activities. Recess offers an opportunity for children to be physically active, interact with friends in an unstructured environment, spend time outside in nature, and most importantly, an opportunity to play which is recognized as one of the rights of being a child [2]. Across Canada, there are different structures for these recess breaks, but students typically get 40–50 min of recess per day. For example, a school might have recess twice a day, with each break lasting around 25-min, while other schools have three recess breaks with a 15 min break in the morning and afternoon and a 1 h break for lunch and recess [3,4]. In some instances, schools have created policies, practices, or procedures to modify recess to be indoors during extreme weather, i.e., high heat, extreme cold, wind, and rain to potentially prevent sickness or injury. Moving recess inside could inhibit students from reaping the full benefits of recess as they will no longer have access to outdoor space which limits access to nature and larger areas for movement and play. Accordingly, this research aims to explore the policies, practices, or procedures of schools regarding weather and recess in the Canadian context.

Recess has consistently been identified as important for children’s health and well-being as it can increase physical activity and allow for social and emotional development [5]. Currently, 65% of Canadian children fail to meet the recommendation of achieving 60 min of moderate-to-vigorous physical activity as outlined in Canada’s 24 h movement guideline [6]. Increasing the number of children that meet this recommendation is vital as physical activity is related to a significant number of physical, mental, and social benefits [7,8,9]. During outdoor recess, children spend almost 40% of recess in moderate-to-vigorous activity and almost 30% in light activity, with children spending their recess playing on the equipment, organized sports and activities, active and chasing games, playground games, exploring nature, and rough and tumble play [10,11]. During outdoor recess, children spend time in nature which is important for children’s development and well-being [12,13]. In some instances, schools have even begun to alter their playground from metal and plastic jungle gyms to more naturalized playgrounds [14]. Time spent in nature and these naturalized playgrounds have been related to positive environmental attitudes, a greater connection to place, improved social and emotional regulation, and enhanced motor fitness and well-being [15]. Finally, children are allowed to play with friends during recess in a less structured environment than within the school. This unstructured play is essential for social, cognitive, and emotional development as children interact with peers and have to solve problems, negotiate, share and communicate [16,17]. If recess is modified to be indoors, children may lose many opportunities to be active, spend time in nature, and develop social and emotional skills.

Given the benefits of outdoor recess for children and youth, it is imperative to understand the policies, practices, and procedures that are currently used to determine if recess is indoors or outdoors. The purpose of this research study is to explore the different policies, practices, and procedures that are used on weather and recess in the Canadian context. Understanding this will allow us to glean insight into how often outdoor recess is modified to be indoors, determine the variability of the policies in Canada, and explore how this may impact children’s activity.

## 2. Methods

Data were gathered from two separate sources. First, a list of all schools in a particular province were obtained from each province’s education website. If the list was unavailable, a search engine (i.e., google) was used to find a list of all schools in a province. For each province, five randomly selected school websites were searched for school weather and recess policies, practices, or procedures. If a website did not have this information, the school was not contacted or retained for the study. This would potentially yield data from 50 schools across Canada. A standardized data extraction template was created to extract weather and school policy, practice, and procedure data. The template included province, weather policy, practice, or procedure (i.e., for what weather conditions recess is modified) and directives for cancellation of outdoor recess (i.e., additional physical activity time). One researcher (PC) independently extracted data, and a second researcher (BB) confirmed the results.

Second, key informant interviews were completed with schoolteachers and administrators over Zoom (Zoom Video Communication, 2022). Schoolteachers and administrators were selected using a purposive and snowball sample based on their experience working in elementary schools in various Canadian provinces. Participants were contacted by recommendations through professional teaching organizations, and then participants were asked if they knew someone else who might be interested. Selected participants did not represent schools from the policy scan. Semi-structured interviews were developed to illicit conversations on school weather and recess policies, practices, or procedures. The interview guide was based on academic and grey literature on school and weather policies [18]. The guide was piloted with an elementary school teacher to ensure the clarity of the questions. Each interview lasted between 30–45 min. Before the interview, a letter of information was sent to each participant, and each participant provided informed oral consent before participating in the study. The questions analyzed for this study included general information questions, i.e., current position, time in teaching or education, primary grades taught, gender identity, and questions about school weather policies. For example, i.e., “in your experience have the schools you taught at had a written policy specifying under what conditions outdoor recess is to be cancelled, moved inside, or modified (i.e., decrease in length)?”. Depending on the answer to this question, a series of questions were asked about the policy and a question was asked about what the participant thought needed to be included in a school weather policy. Finally, participants were given the opportunity to add any other thoughts they had about school weather policies. Immediately after each interview, detailed field notes were taken that described the data quality, the moderator’s impression of the interview, and notes about the content and structure of the interview. Each interview was transcribed verbatim. Originally, the research team aimed for 13 in-depth interviews, but we started seeing repeated ideas after interview 8. Ethics for this project was submitted and approved by the University of Winnipeg Research Ethics Board (IRB Protocol# HE16949).

For both the school weather policy, procedure, and practice scan and the semi-structured interviews we used a qualitative content analysis to understand key themes from the data. PC coded for different weather occurrences that led to a cancellation in recess and then categorized any other information relevant to the research purpose. To ensure trustworthiness, a second researcher (BB) confirmed the findings.

## 3. Results

Policies were extracted from 14 different schools across Canada and are presented in Table 1. Of note, thirty-six schools did not have a policy listed on their school website. Policies in the 14 schools fell into 4 categories, (1) cold, (2) heat, (3) precipitation (i.e., rain, snow), (4) air quality. Cold temperature cut-offs were described in 13 school policies, with temperatures ranging from −20 to −40 °C for complete time in indoor recess. Some schools indicated that students would be allowed outside for a few minutes at certain temperatures and then return inside. Four schools stated that they had a policy on precipitation but did not include specific measurements or quantifiable indicators. Two schools had heat considerations, one school had an air quality cut-off, and some schools did mention heat or smog as something to consider.

### Interviews

In total, seven teachers and three administrators participated in the interview. Sample information can be found in Table 2.

Participants were asked if schools they had taught at had a written policy specifying under what conditions outdoor recess was modified to be inside. Participants described both formal written policies, and others described more informal policies. The formal written policies included specific temperature cut-offs and, in some instances, precipitation, heat, and wind. Regarding informal policies, one participant said:

“I gather it’s something that should be provided, but there isn’t a set policy. It’s definitely not on the policies website for the school board, and both my principal and the vice principal didn’t have it readily available. It was just more of a subjective thing and a general kind of guideline that had been discussed at vice principal and principal meetings.” (Interview 9)

When teachers or administrators discussed formal and informal weather policies, they all had slightly different descriptions of what would constitute indoor recess during precipitation. For example, one participant said:

“Yeah, it’s not on a measurement. If it’s like spitting; they’ll be like, okay, you can go outside for a certain amount of time unless it starts to rain more, but it’s not a specific number. It wouldn’t be like three centimetres they don’t give us a number it’s just that if the kids are going to get wet, they bring them all in.” (Interview 8)

When implementing the policies, some key informants discussed that rules were followed to the number. At the same time, other participants mentioned that there was more give-and-take between teachers, supervisors, and administrators. One participant said that:

“There is a rumored guideline that our school board follows but there seems to be discrepancies between different schools. From what we hear with our colleagues at other schools, our school seems to be pretty stringent on not sending the students out in any inclement weather, and we know that some schools, basically say ensure your child is dressed properly, we will be going out in any type of weather.” (Interview 2)

Finally, teachers and administrators were asked if they had any suggestions or ideas for what school policies on weather should include. Some teachers suggested that they were happy with the current official or unofficial policies. Still, some teachers or administrators wanted greater clarity on the background and implementation of the policy, as described by one informant.

“Yeah, I would want to know how that number was derived. If that was in consultation with a health unit or a medical professional. I don’t know how that was derived, but I would like to know… So just to maybe understand it, the reasoning behind it. ” (Interview 5)

* Direct quotes have been edited for readability.

## 4. Discussion

The purpose of this study was to explore the different policies, practices, and procedures that are used on weather and recess in the Canadian context. A review of school websites and interviews with key informants found a large portion of schools do not have an outwardly facing official weather and recess policy. Of the schools with a weather and recess policy, there is variability in temperature and precipitation cut-offs. Finally, some teacher’s discussed a lack of understanding of the specifics of the weather policy. This information can be used to help advocate for more evidence-based policies and practices that keep children outside, active, and engaging with peers during recess.

Temperature was the main feature of school weather and recess policies. Temperature cut-offs ranged from −20 to −28 or +35 degrees Celsius for modification to indoor recess. Once this threshold has been met opportunities for outdoor recess and the associated benefits of physical activity, certain types interpersonal interactions, and time in nature would either be greatly reduced or completely taken away. The cut-off of −27 aligns with moderate risk level according to Environment Canada [33]. Some schools did allow students outside in colder temperatures to get some fresh air or a quick walk and right back inside. Allowing students outside for a brief period, i.e., walking to the fence and back, could be beneficial as it would reduce sedentary time, increase time spent in nature, and potentially allow students to interact with peers from other classes [21,26]. Future research is needed to understand if these micro-breaks benefit children’s health and well-being and how to make best use of shorter outdoor periods. These findings could support policies that guarantee students get a certain amount of time outside per day safely.

Precipitation was only found in four school weather and recess policies but was identified as a reason that recess was modified to be indoors by all informants in the interviews. This finding suggests that guidelines are being formed at the school level, typically by administrators or a combination of administration and teachers. Future research is needed to understand when recess is being modified to be inside due to precipitation and, more specifically, rain. For example, in Vancouver, from 1971–2000 there were, on average, almost 170 days of precipitation, with July and August, the two months that students have off from school, having the least amount of rain days on average [34]. With almost 170 days of precipitation, there is a potential for a large portion of school recesses to be modified to be indoors. A study from the UK found that on days with rain, children got the least amount of physical activity during recess and suggested indoor opportunities for physical activity might be an effective solution to prevent the decline in physical activity [35]. However, many schools are dealing with overcrowding, and some schools had to remove that opportunity due to COVID-19. Therefore, it might be difficult to offer indoor opportunities for physical activity for all students. Future research is needed to understand why children are less active on rainy days and ways to mitigate these barriers.

Another key finding is that there were few policies found addressing air quality and heat. This is an area where more discussion and policy development are needed. Due to climate change, extreme weather events are becoming more frequent. For example, British Columbia has seen unprecedented high heat and wildfires which impact air quality [36,37]. Children are especially susceptible to the health impacts of such environmental conditions [38]. Therefore, creating evidence-based plans and policies that protect children from such extreme weather conditions, while still supporting the benefits children receive from outdoor recess, is an important area of future inquiry. Without such plans and policies ad hoc practices may be used which could limit physical activity, social interaction, and time in nature and not provide the best opportunities for children and youth.

What can be concluded from the teachers and administrators and information on school websites is there is variation in the policies, practices, and procedures used to govern school recess. Of the policies, practices, and procedures that do exist they typically only provide guidance on when to modify and not what do to support children during these modifications. In Ontario, a Canadian province, there is no standard school and weather policy, but there are standard policies for daily physical activity, food and beverages, asthma, concussions, and protected time for specific subjects, among many others [39]. Although implementing certain school-based policies has had mixed levels of success, creating a provincial or territorial weather and recess policy would bring attention to this area [40]. Evidence-based guidelines should also be created for all weather conditions to ensure that recess is not being modified indoors without cause and that schoolyards are designed to encourage outdoor play in extreme weather conditions.

Several limitations need to be considered when interpreting these results. First, we collected data from school websites which might not contain the most recent recess and weather policy. Second, a purposive sample was used and there is a potential that the sample was biased since the primary researcher made subjective decisions on choosing who would participate in the study. To prevent this bias, the researcher selected teachers from different provinces, various years of experience, and different subject specialties. Finally, we extracted data on school location, but determined that the purpose of this study was to explore policies across Canada and not compare policies by province. Regardless of where a school is located, they will face extreme weather, and with more frequent weather extremes, it would benefit school staff and children to consider numerous weather conditions.

## 5. Conclusions

The results of this study suggest that many schools lack a school and recess weather policy and individual schools create their own policies without evidence for their local practices. Provinces need to establish policies, practices, and procedures that are reflective of their weather systems and provide scenarios for school administrators, so they are able to implement the policy effectively. With weather patterns having the potential to modify recess to be indoors and recess providing countless benefits, it is necessary that this area be further explored, and evidence-based policies are created to ensure children can get at least some of the benefits of recess.

## Figures and Tables

**Table 1:** School weather and recess policies from 14 Canadian schools.

School	Cold	Heat	Precipitation	Air Quality
School 1 [19]	NA	NA	NA	Air quality health index 4–6 modify for at-risk participants 7 and above all indoor activities moved inside
School 2 [20]	−25 and wind chill must be considered indoor recess	NA	Very stormy indoor recess	NA
School 3 [21]	−23 and below indoor recess	NA	Raining heavy indoor recess	NA
School 4 [22]	−25 to −40 modified (a few minutes outside) −40 with windchill indoor recess	NA	NA	NA
School 5 [23]	−27 with windchill indoor recess	NA	NA	NA
School 6 [24]	−22 to −24 with windchill students can warm-up inside −25 with windchill indoor recess	NA	NA	NA
School 7 [25]	−28 indoor recess with windchill	NA	NA	NA
School 8 [26]	-25 to -28 with windchill (a few minutes outside) −28 with windchill indoor recess	NA	NA	NA
School 9 [27]	−27 with windchill indoor recess	NA	NA	NA
School 10 [28]	−20 with windchill consider indoor recess	Hot/humid weather	Different types of precipitation (i.e., rain, snow) are listed for consideration for indoor recess.	NA
School 11 [29]	−20 to −28 modification to shortened recess −28 indoor recess	NA	Different types of precipitation (i.e., rain, snow) are listed for consideration for indoor recess.	NA
School 12 [30]	−25 to −28 indoor recess	NA	NA	NA
School 13 [31]	−20 with windchill indoor recess	Hot/humid weather	NA	NA
School 14 [32]	−21 to −27 serious consideration should be given if it is appropriate to send students outside −27 with windchill indoor recess	NA	NA	NA

All temperatures in °Celsius. NA = Not available.

**Table 2:** Sample information for the interview participants.

Sample Characteristic	*n*
Position	
Teacher	7
Administration	3
Gender	
Male	2
Female	8
**Sample Characteristic**	**Average/SD**
Years in education	10/5 *

SD = Standard Deviation. * Average years in education is based on 9 participants. One participant used a qualitative descriptor that cannot be translated to a numerical measure.

## Data Availability

The data presented in this study are available on reasonable request from the corresponding author.
